# Mathematical models of C-type and N-type inactivating heteromeric voltage gated potassium channels

**DOI:** 10.3389/fncel.2024.1418125

**Published:** 2024-10-08

**Authors:** Kees McGahan, James Keener

**Affiliations:** Math Department, University of Utah, Salt Lake City, UT, United States

**Keywords:** mathematical modeling, ion channel kinetics, heteromeric potassium ion channels, *K*_*V*_ channels, *K*_*v*_1 channels

## Abstract

Voltage gated potassium channels can be composed of either four identical, or different, pore-forming protein subunits. While the voltage gated channels with identical subunits have been extensively studied both physiologically and mathematically, those with multiple subunit types, termed heteromeric channels, have not been. Here we construct, and explore the predictive outputs of, mechanistic models for heteromeric voltage gated potassium channels that possess either N-type or C-type inactivation kinetics. For both types of inactivation, we first build Markov models of four identical pore-forming inactivating subunits. Combining this with previous results regarding non-inactivating heteromeric channels, we are able to define models for heteromeric channels containing both non-inactivating and inactivating subunits of any ratio. We simulate each model through three unique voltage clamp protocols to identify steady state properties. In doing so, we generate predictions about the impact of adding additional inactivating subunits on a total channel's kinetics. We show that while N-type inactivating subunits appear to have a non-linear impact on the level of inactivation the channel experiences, the effect of C-type inactivating subunits is almost linear. Finally, to combat the computational issues of working with a large number of state variables we define model reductions for both types of heteromeric channels. For the N-type heteromers we derive a quasi-steady-state approximation and indicate where the approximation is appropriate. With the C-type heteromers we are able to write an explicit model reduction bringing models of greater than 10 dimensions down to 2.

## Introduction

Potassium channels are an evolutionarily conserved set of membrane spanning proteins (Harding et al., 2022; MacKinnon, 2003). While the singular role of these channels is to permit the flux of potassium ions across the impermeable cell membrane, this seemingly simple function is responsible for a wide range of complex physiological processes. In excitable cells, such as neurons, muscle cells and pancreatic β-cells, the primary purpose of potassium channels is to change the shape of, or terminate, action potentials. For non-excitable cells, potassium channels are responsible for functions like volume regulation, maintaining cell shape and electrolyte balance, and neurotransmitter release (Barfield et al., 2005; Ghatta et al., 2006; Hoshi et al., 1990; Miller, 2000).

Although some of these channels are constitutively open, the majority are gated. Gated potassium channels are divided into three main subsets: ligand gated (*K*_*ir*_), calcium activated (*K*_*Ca*_), and voltage gated (*K*_*V*_) channels (Coetzee et al., 1999; Harding et al., 2022). Contained within each of these classes are a number of unique channels which all have a distinct set of kinetic properties. The *K*_*V*_ family has roughly 40 genes, which each encode a single α-protein subunit. A total of 4 of these subunits are required to form a functional tetrameric *K*_*V*_ channel (Harding et al., 2022; Cordeiro et al., 2019). Experimentally it has been shown that these 4 subunits need not be identical. When they are identical, the channels are referred to as homomeric channels, but in the cases when more than 1 subunit type is present, a heteromeric channel is formed (Cordeiro et al., 2019; Al-Sabi et al., 2013). The existence of these heteromeric *K*_*V*_ channels is a relatively recent discovery, and has lead researchers to start investigating heteromeric channel properties in relation to the homomeric channels comprised of similar subunits.

Through a variety of experimental techniques, including mathematical modeling, researchers have begun developing a clearer picture of the role and function of heteromeric *K*_*V*_ channels. Working with cone snail toxins, Cordeiro et al. (2019) demonstrated that these snails have naturally occurring compounds designed to target heteromeric *K*_*V*_ channels with specific combinations of subunits in their prey. Al-Sabi et al. (2013) showed using concatemeric *K*_*V*_1.1/*K*_*V*_1.2 constructs that heteromeric channels have activation open probability curves that lie between those of the related homomeric channels. Furthermore, they found a nonlinear shift in these open probability curves as additional *K*_*V*_1.2 subunits were included in the heteromeric concatemers. In previous modeling work, we replicated the Al-Sabi et al. (2013) observations about heteromeric channels with a mechanistic Markov model. We also showed the model's predictive ability in relation to a number of cDNA expression experiments (McGahan and Keener, 2022). While these studies have elucidated certain features of heteromeric channels, the emphasis has been on studying *K*_*V*_ channels with little to no inactivation.

Voltage gated potassium channel inactivation has been shown to take two forms: N-type and C-type inactivation. N-type inactivation is believed to be a voltage insensitive process by which a single “tethered ball” segment at the N-terminal of a subunit binds to, and occludes, the open channel pore (Holmgren et al., 1996; Sukomon et al., 2022; Hoshi et al., 1990; Miller, 2000). Meanwhile, C-type inactivation is believed to be an internal conformational change happening either near the mouth of the channel, or in the selectivity filter (Tan et al., 2022; Cuello et al., 2010; Hoshi et al., 1990; Miller, 2000). These inactivation mechanisms have been studied in homomeric *K*_*V*_ channels using techniques such as cryo–electron microscopy, mathematical modeling, and deletion/mutation experiments (MacKinnon et al., 1993; Hoshi et al., 1990; Miller, 2000; Al-Sabi et al., 2011). One prominent modeling study conducted in 2011 by Bett et al. (2011), used mutated *K*_*V*_1.4 channels to derive and fit Markov models for N and C type inactivating homomeric channels.

In spite of this work, and the plethora of experimental techniques and computing power available, the understanding of N-type and C-type inactivation in heteromeric channels is incomplete. What is known regarding heteromeric channel inactivation, is that a single subunit from an inactivating homomeric channel is sufficient to confer inactivation kinetics in a heteromer. By combining specific mutations with the effects of a scorpion toxin, MacKinnon et al. (1993) were able to demonstrate the existence of this property in Shaker potassium channels. Related to this work, Hashimoto et al. (2000) created mutated *K*_*V*_1.4 channels with different numbers of tethered inactivation N-Balls to explore the impact of each additional ball on the homomeric channel. Much later, in 2011, Al-Sabi et al. (2011) showed that the N-type inactivation prevention domains of *K*_*V*_1.6 subunits could be used to reduce, or eliminate, the effects of a single *K*_*V*_1.4 subunit's N-type inactivation in a heteromeric concatemer. Yet to be addressed experimentally, or via modeling, are questions surrounding the impact of including additional N-type or C-type inactivating subunits and the resulting heteromeric channel's overall kinetic properties. In particular:

1. What mathematical model structure for the homomeric channels is necessary to ensure a heteromeric channel with single inactivating subunit will inactivate?
2. Do more N-type/C-type subunits cause greater level of channel inactivation and slow the rate of recovery?
3. Is the relationship between number of N-type/C-type subunits and the level of inactivation linear, or is it nonlinear as was observed with the activation curves of *K*_*V*_1.1/*K*_*V*_1.2 (Al-Sabi et al., 2013)?
4. Are there distinguishable differences between the impact that C-type versus N-type inactivating subunits have on a heteromeric channel?

Building upon previous experimental and modeling results, with the goal of addressing these types of questions, we propose novel, biophysically detailed mathematical models for heteromeric channels with either N-type or C-type inactivating subunits. We begin by briefly outlining the model used for non-inactivating, homomeric and heteromeric voltage gated potassium channels (McGahan and Keener, 2022). Then, using the (Bett et al., 2011) models as a guideline, we construct homomeric N-type and C-type inactivating channel models. Finally, for both N-type and C-type inactivation, we describe how to extend the homomeric models to heteromeric channels, show the response of every heteromeric and homomeric model to a series of complex voltage clamp protocols, and outline any relevant model reductions.

## Methods

### Voltage gated potassium channels: no inactivation

To model a non-inactivating, homomeric, voltage gated potassium channel, we assume there are 4 identical and independent subunits, each of which can be in either the open or closed conformation. Furthermore, all 4 subunits must be in the open state to have a conducting channel, and only one subunit can change state at a time. With these assumption, the probability of having *i* subunits in the open state can be described with the master equation:


(1) $$\begin{array}{l} \frac{dp_{i}}{dt}=\left(4-i+1\right)\alpha p_{i-1}+\left(i+1\right)\beta p_{i+1}-\left(4-i\right)\alpha p_{i}-i\beta p_{i}, \end{array}$$

where α and β are the rates of a single subunit transitioning from the closed to the open state and vice versa. This model has classically been used to describe channels of this type, dating as far back as the first mathematical description of potassium channels by Hodgkin and Huxley (1952). The model detailed in the groundbreaking work by Hodgkin and Huxley has been shown to be an exact one dimensional model reduction of this more general Markov scheme (Keener, 2009, 2010). Using the same assumptions made for a homomeric channel, in earlier work we detailed how to model a heteromeric channel lacking significant inactivation kinetics (McGahan and Keener, 2022). In that work, we described both a general Markov model and detailed a 2 dimensional model reduction for each possible subunit ratio. These reduced systems of differential equations were shown to be globally attracting stable invariant manifolds, meaning the reductions were exact and replicate the behavior of the full system as time is taken to infinity (Keener, 2009, 2010).

The work below addresses homomeric and heteromeric channels possessing inactivation kinetics. For both N-type and C-type inactivation, we present and justify a homomeric channel model, describe how to extend these models to heteromeric channels, show the response of every model to a series of different voltage clamp protocols, and outline any relevant model reductions.

### Voltage clamp simulations

**Figure 1:**
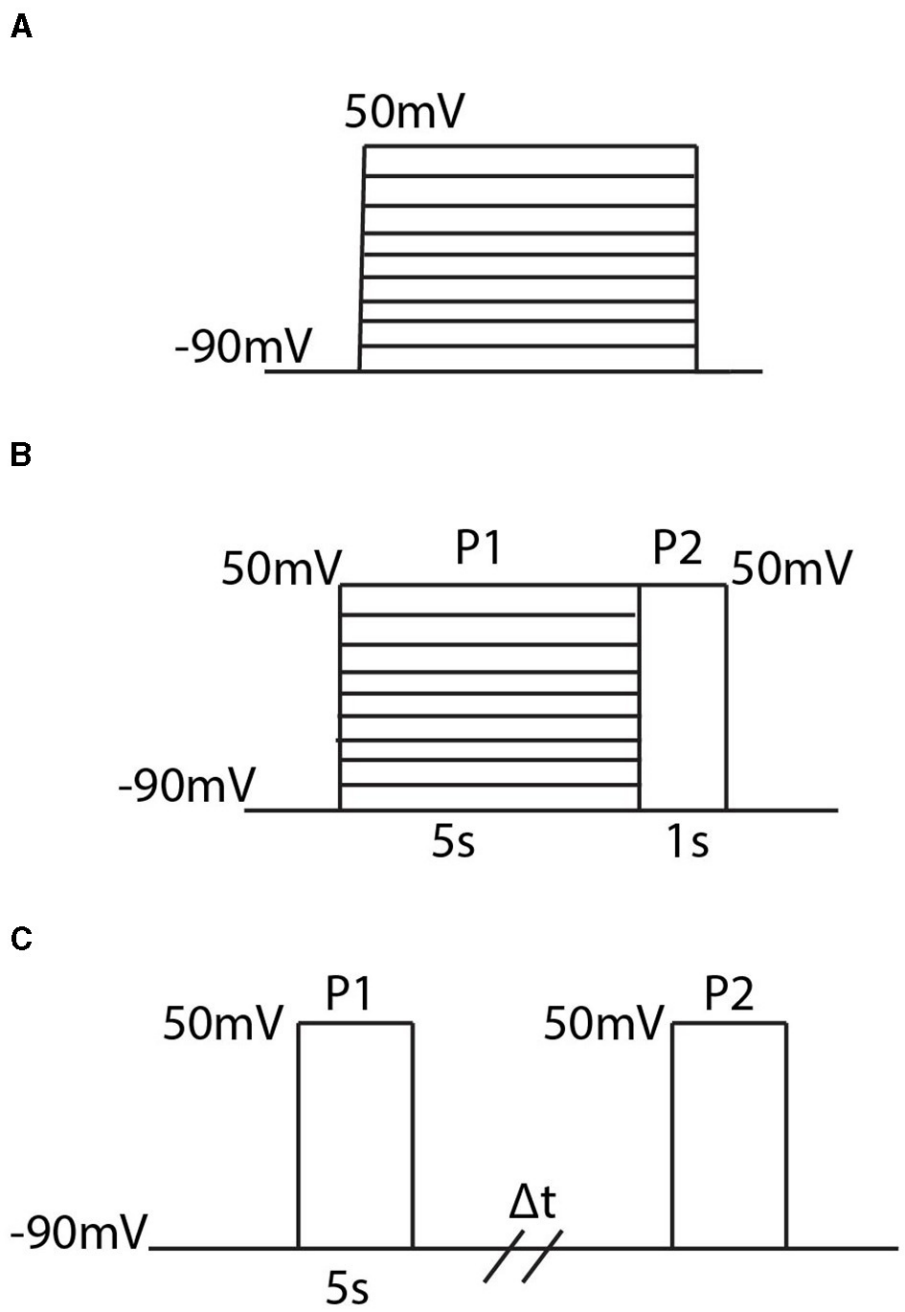
Diagrams of the three different voltage clamp experiments used for model fitting and analysis. **(A)** The activation protocol begins by holding the membrane potential at −90 *mV* and then, in increments of 10 *mV*, steps and holds the membrane potential at some new voltage between −90 *mV* and 50 *mV*. **(B)** The inactivation protocol steps the voltage from −90 *mV* to a new voltage between −90 *mV* and 50 *mV* where it is held for 5s. Then regardless of the initial voltage step, the voltage is increased to 50 *mV* and held there for 1 second. **(C)** The recovery protocol performs 2 pulses P1 and P2 which are separated by a variable time duration Δ*t*. In each pulse the membrane potential is stepped from −90 *mV* to 50 *mV*.

With each homomeric and heteromeric model, for fitting and analysis, we simulate the model using the three different voltage clamp protocols whose schematics are shown in Figure 1.

In order, the voltage clamp protocols are referred to as the activation protocol (Figure 1A), the inactivation protocol (Figure 1B), and the recovery protocol (Figure 1C). The activation protocol fixes the membrane potential at −90 *mV* and then, by multiples of 10 *mV*, steps the membrane potential to some new voltage value. From this protocol, 2 pieces of information are extracted: the maximum open probability attained, and the time constant, τ. By tracking the probability of the channel being in the open state, *O*, during this simulation, the maximum open probability is the maximum value *O* attains during the activation protocol in response to the value the voltage was stepped to. To remain consistent with previous experimental work, and the relevant experimental data used for model fitting, the second piece of information extracted from the activation protocol, for only the homomeric channels, is a time constant τ. Both the methodology for calculating τ, and the justification behind only calculating τ for homomeric channel model fitting, can be found in McGahan and Keener (2022). As more experimental data is made available, the fitting techniques and analyses could easily be adjusted to incorporate the entire time course traces seen during these protocols.

The inactivation protocol (Figure 1B) provides steady state inactivation information about the channel. In this protocol, the channel is exposed to 2 different pulses *P*1 and *P*2. The *P*1 pulse is a replica of the activation protocol, but is made to last for 5 seconds. Then, regardless of the voltage the channel is set to, during *P*2 the voltage is raised to 50 *mV*. The recorded output of this protocol divides the maximum value of *O* during *P*2, by the maximum value found during *P*1. This resulting ratio is then plotted in response to the value the voltage was stepped to during *P*1.

The last protocol is the recovery protocol (Figure 1C). This experiment begins with the first pulse *P*1 by stepping the fixed potential from −90 *mV* up to 50 *mV* and holding the voltage there for 5 seconds. After these 5 seconds, the voltage is returned to −90 *mV* for a variable time duration Δ*t*, until it is forced back to 50 *mV* during *P*2. Similar to the inactivation protocol, the value of interest is the maximum probability of the open state *O* seen during *P*2 divided by the value seen during *P*1. This ratio is then plotted in response to the varying time duration Δ*t* to get a metric of how long it takes the channel to return to the closed, non-inactive, state.

## Results

### Homomeric N-type inactivating channels

Here we detail a Markov model for N-type inactivating channels. Work by Bett et al. (2011) mutated a *K*_*V*_1.4 channel to reduce C-type inactivation capabilities resulting in a channel with primarily N-type inactivation. Using this mutated channel, a 6 state Markov model was proposed and fit for a homomeric N-type inactivating *K*_*V*_1.4 channel by Bett et al. (2011). This Markov model, with a minor adjustment for clarity as we extend to heteromeric channels, is given below:

Similar to the homomeric channel lacking inactivation described above, there are assumed to be four independently gated subunits all of which must be in the open conformation to have a conducting channel. From the open state *O*, the subunits can close again, or a single lipophilic N-terminal “ball” region from one of the subunits can bind to the channel opening, thereby transitioning the channel to an inactive state (Holmgren et al., 1996; Sukomon et al., 2022). As there are 4 subunits, each containing an N-terminus, we write this transition rate from *O* to *I* as 4*a*_*I*_. However, as the unbinding rate of the single bound N-terminal ball is independent of the number of subunits, the transition rate from *I* to *O* is written as *b*_*I*_. The differential equations describing this model are:


(2) $$\begin{array}{l} \frac{dC_{i}}{dt}=\left(4-i+1\right)a_{1}C_{i-1}+\left(i+1\right)b_{1}C_{i+1}\\ -\left(4-i\right)a_{1}C_{i}-ib_{1}C_{i},\;i\leq 3\\ \frac{dO}{dt}=a_{1}C_{3}-4b_{1}O-4a_{I}O+b_{I}I\\ \frac{dI}{dt}=4a_{I}O-b_{I}I. \end{array}$$

### Heteromeric N-type inactivating channels

**Figure 2:**
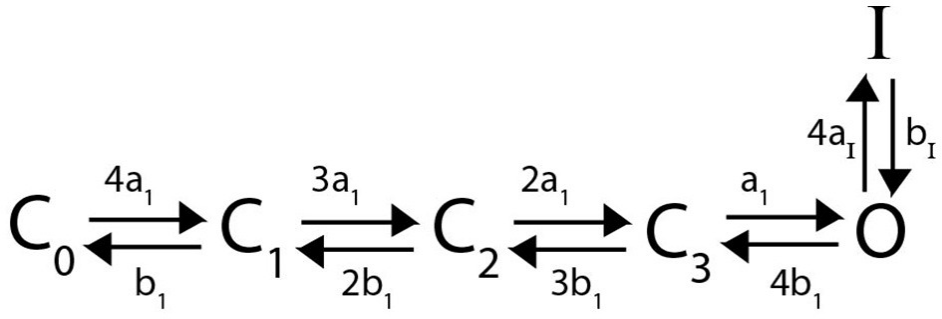
A Markov model diagram for a homomeric N-type inactivating channel. The states *C*_*i*_ denote the probability of having *i* subunits in the open state. The probability of being in the conducting state is given by *O*, where all 4 subunits are in the open state. *I* is the probability of the channel having an N-ball bound to the pore and being in the inactive state. The kinetic rates *a*_1_, *b*_1_ give transitions between a single subunit going from closed to open state and vice versa. The rate of a single N-ball binding to the pore is *a*_*I*_ and the unbinding is given by *b*_*I*_.

**Figure 3:**
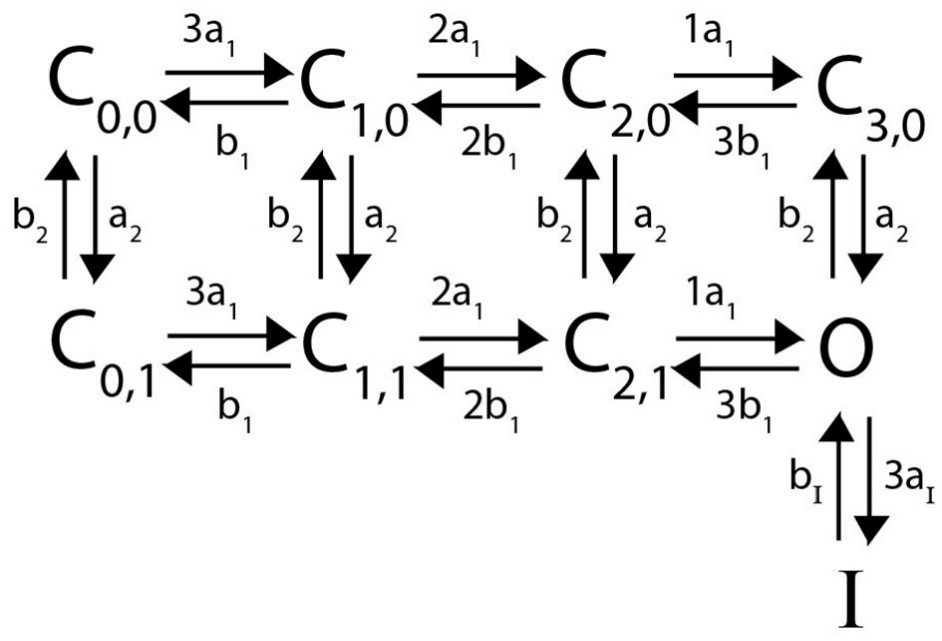
A Markov model diagram for a heteromeric channel with 3 N-type inactivating subunits and 1 non-inactivating subunit. The state *C*_*i*_, *j* is the probability of having *i* N-type inactivating subunits in the open state and *j* non-inactivating subunits in the open state. The states *O, I* and rates *a*_1_, *b*_1_, *a*_*I*_, *b*_*I*_ have identical meaning to that of Figure 2. The rates *a*_2_ and *b*_2_ give the opening and closing transitions for the non-inactivating subunit.

Following in the spirit of the proposed model structure by McGahan and Keener (2022), we assume that a heteromeric channel still requires 4 activating gates, one per subunit type. To account for the N-terminal “ball” binding to the channel pore in a heteromeric channel, there is still only a singular inactive state as in Figure 2. However, the transition rate from *O* to *I* now becomes *na*_*I*_, where *n* is the number of N-type inactivating subunits in the heteromer with 4*a*_*I*_ the transition rate in the homomeric channel. A general example Markov model scheme for a channel with 3 N-type inactivating subunits, and 1 non-inactivating subunit is given in Figure 3.

**Figure 4:**
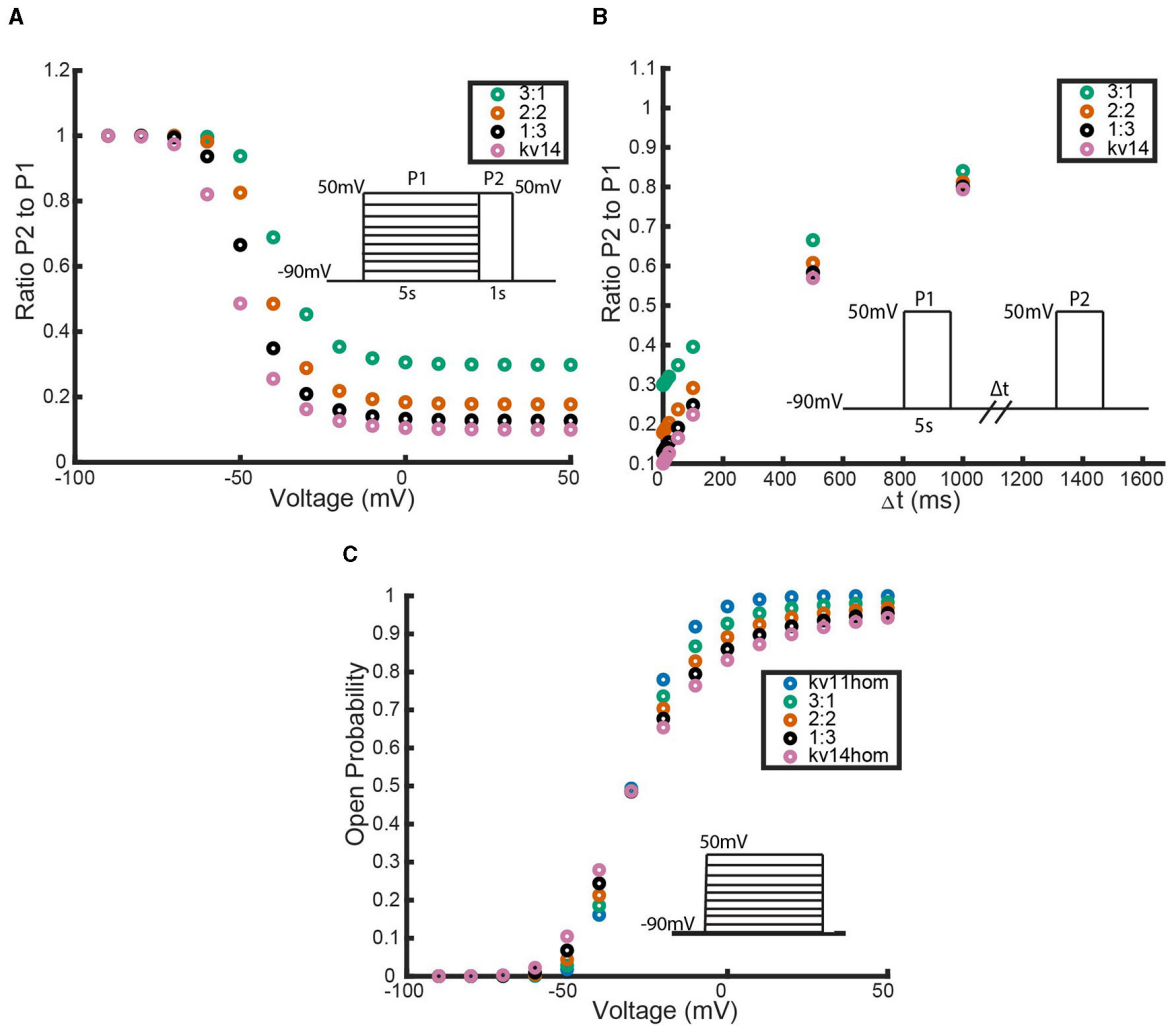
Responses of each heteromeric and homomeric channel with *K*_*v*_1.4[*K*532*Y*] N-type inactivating subunits and *K*_*V*_1.1 non-inactivating subunits to the three voltage clamp protocols of Figure 1. **(A)** The ratio of the maximum open probability of the channel seen during P2 compared to P1, plotted against the voltage stepped to during P1 based on the inset inactivation protocol. **(B)** The ratio of the maximum open probability of the channel seen during P2 compared to P1, plotted against the time duration, Δ*t*, between P1 and P2, based on the inset recovery protocol. **(C)** The maximum open probability of the channel after the voltage step, plotted against the voltage the channel was stepped to. Data point colors are: pink (4 *K*_*v*_1.4[*K*532*Y*] subunits), black (1 *K*_*V*_1.1 to 3 *K*_*V*_1.4[*K*532*Y*] subunits), orange (2 *K*_*V*_1.1 to 2 *K*_*V*_1.4[*K*532*Y*] subunits), green (3 *K*_*V*_1.1 to *K*_*V*_1.4[*K*532*Y*] subunits) and blue (4 *K*_*V*_1.1 subunits).

To examine the predictive outputs of this model scheme, we consider heteromeric complexes forming with *K*_*V*_1.1 and *K*_*v*_1.4 subunits (Harding et al., 2022; Bett et al., 2011). In an attempt to accurately reflect the proper channel (and subunit) kinetic properties, forward and backward activating and inactivating rates are taken from Markov models that have been experimentally fit using voltage clamp data. The transition rates for the *K*_*V*_1.1 subunits are drawn from Masoli et al. (2015). For *K*_*V*_1.4 channel kinetics, we used the model fit by Bett et al. (2011) to voltage clamp data for *K*_*v*_1.4[*K*532*Y*] mutated channels which are known to possess N-type, but lack C-type, inactivation. Parameterizing the subunits in this manner, we took each possible subunit combination and exposed the corresponding heteromeric and homomeric channels to the voltage clamp protocols described in Figure 1. The results of these simulated experiments are shown in Figure 4. We emphasize here that although the simulations here are centered around *K*_*V*_1.4 and *K*_*V*_1.1 heteromeric channels, the methodology and proposed model scheme is widely applicable provided the channels follow the identical and independently operating subunit assumptions.

As response of the *K*_*V*_1.4[*K*532*Y*] and *K*_*V*_1.1 homomeric channels to the activating protocol are similar, this experiment does little to distinguish the heteromeric channels. However, in both the inactivation protocol and the recovery from inactivation protocol a trend becomes clear. The first observation to be made is that any number of *K*_*V*_1.4 subunits confer inactivating kinetics upon the channel. Furthermore, as the number of *K*_*v*_1.4[*K*532*Y*] subunits increases, the level of inactivation increases and the rate of recovery decreases. Additionally, these trends are nonlinear. That is, at higher voltage values, there is a noticeably larger change in probability curves moving from the 3:1 heteromer to the 2:2 heteromer, than transitioning between the probability curves of the 1:3 and 0:4 channels. This phenomenon is replicated in the recovery from inactivation curves as well. By increasing the number of *K*_*v*_1.4[*K*532*Y*] subunits, the channel takes longer to recover from inactivation, with the first *K*_*v*_1.4[*K*532*Y*] subunits having a larger impact than the last.

### N-type inactivating model: QSS approximation

The largest of these heteromeric models is the 2:2 heteromeric channel consisting of 10 ordinary differential equations. For models of this scale it is useful to find ways to reduce dimension, particularly if the models are to be incorporated into larger neuron models or used to simulate more complex protocols for extended time intervals. It has been shown that for homomeric and heteromeric channels without inactivation kinetics there exists a one (or two in the case of heteromeric channels) dimensional, globally attracting stable invariant manifold (Keener, 2009, 2010). In other words, the channel kinetics of the full system can be described by one (or two) differential equations. However, the introduction of the N-type inactivating state *I*, to both the heteromeric and homomeric channel models, complicates the process of finding an invariant manifold to simplify model complexity.

**Figure 5:**
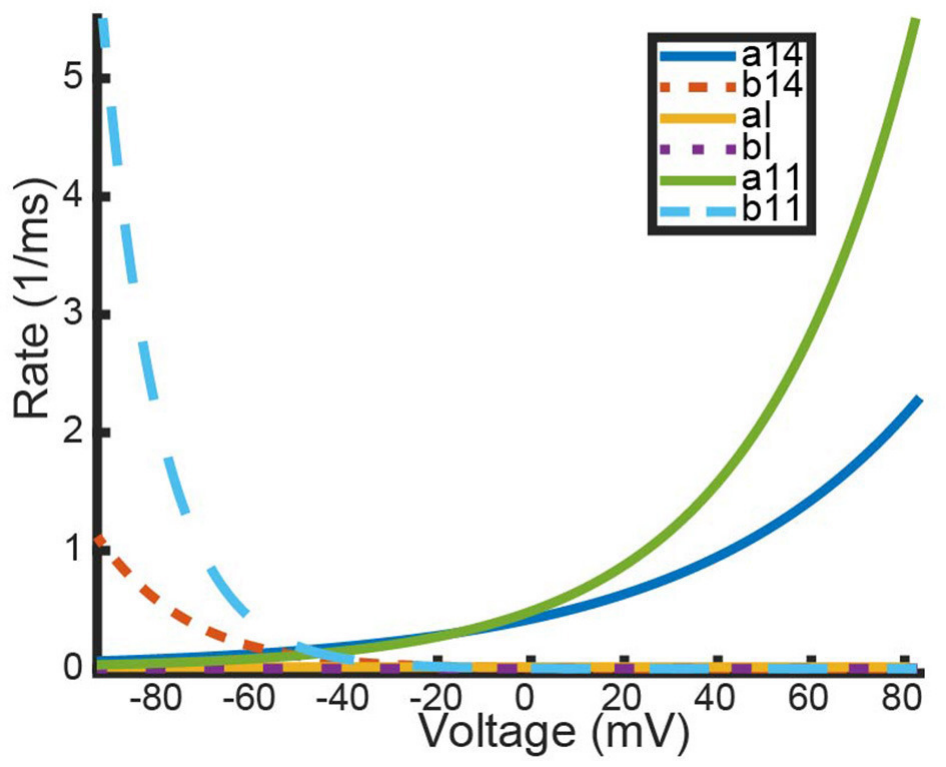
Experimentally fit kinetic transition rates plotted against a given voltage in *mV*. The following rates are plotted: opening and closing rates of *K*_*v*_1.4[*K*532*Y*] subunits (*a*_14_, *b*_14_), opening and closing rates of *K*_*v*_1.1 (*a*_11_, *b*_11_), and the binding and unbinding rates of the N-balls, (*a*_*I*_, *b*_*I*_). Curve color and style are given in the figure legend.

That being said, a second common technique for dimension reduction, is a quasi-steady-state (QSS) approximation (Keener and Sneyd, 2009). This technique of model simplification relies on assuming, or knowing, that some of the kinetic transitions occur much faster than others. Utilizing the kinetic rates for the homomeric *K*_*V*_1.1 and *K*_*v*_1.4[*K*532*Y*] models that were used to generate the results of Figure 4, we show below in Figure 5, that for these Markov models with N-type inactivation, this may be a reasonable assumption.

In Figure 5, it can be seen that for a significant portion of the physiologically relevant membrane potentials, the rates *a*_1_, *a*_2_, *b*_1_, *b*_2_ >> *a*_*I*_, *b*_*I*_, i.e., transitioning between closed states is much quicker than transitioning to the inactive state. Using the *K*_*V*_1.4 homomeric model as an example, we outline the process for using this relationship between the kinetic rates to construct a one dimensional ODE approximation of both the full heteromeric and homomeric *K*_*V*_1.1:*K*_*v*_1.4[*K*532*Y*] Markov models.

Working with the model depicted in Figure 2, the first step in the QSS approximation is to define a new variable *X* = *C*_0_ + *C*_1_ + *C*_2_ + *C*_3_ + *O*. By summing together the ODEs for *C*_0_, *C*_1_, *C*_2_, *C*_3_, *O* we arrive at the ODEs:


(3) $$\begin{array}{l} \begin{array}{c} \frac{dX}{dt}=-a_{I}O+B_{I}I.\\ \frac{dI}{dt}=4a_{I}O-b_{I}I. \end{array} \end{array}$$

To get these ODEs to be functions of only *X* and *I* we must solve for *O* in terms of *X*. This is done by making use of the fast equilibrium assumption that *a*_1_, *a*_2_, *b*_1_, *b*_2_ >> *a*_*I*_, *b*_*I*_ to solve the ODEs of Equation 2 at steady state. Starting with $\frac{dO}{dt}=0$, using the fast equilibrium assumption, the *b*_*I*_*I* and −*a*_*I*_*O* terms go to 0, and we are left with $O=\frac{-a_{1}C_{3}}{4b_{1}}$. The remainder of the ODEs for *C*_0_, *C*_1_, *C*_2_, *C*_3_ can be solved in iteration at steady state to get an expression in terms of *O*. This gives *X* = κ*O*, where, for the homomeric channel, $\kappa =\frac{{\left(a_{1}+b_{1}\right)}^{4}}{a_{1}^{4}}$. Noting that *X* and *I* are probabilities of being in a particular state, we have *X* + *I* = 1. Therefore, we can fully describe the system with the single ODE:


(4) $$\begin{array}{l} \frac{dX}{dt}=-a_{I}N\frac{X}{\kappa }+b_{I}I. \end{array}$$

Here *N* is the number of N type subunits, which for the homomeric channel case gives *N* = 4. Using the relationships *X* = κ*O* and *X* = *C*_0_ + *C*_1_ + *C*_2_ + *C*_3_ + *O*, it is possible to simulate the one-dimensional system, and then back out the value of any of the desired states *C*_0_, *C*_1_, *C*_2_, *C*_3_, *O*.

**Figure 6:**
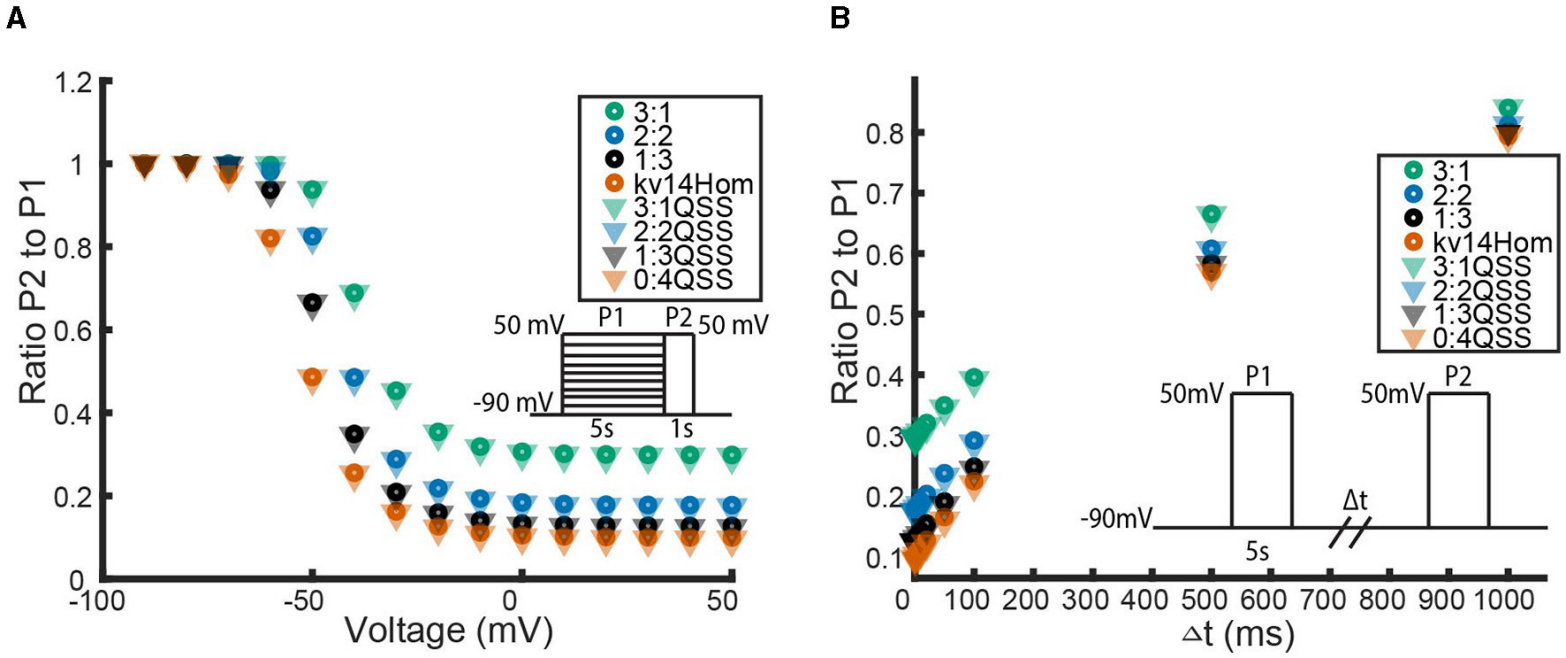
A comparison between the full *K*_*V*_1.1:*K*_*v*_1.4[*K*532*Y*] heteromeric models and the QSS reduced *K*_*V*_1.1:*K*_*v*_1.4[*K*532*Y*] heteromeric models by looking at the channel responses to the inactivation protocol **(A)** and recovery protocol **(B)**. Data point colors correspond to the identical subunit ratio described in Figure 4, with circles the full model data points and triangles the QSS model data points.

For any of the *K*_*V*_1.1:*K*_*V*_1.4[*K*532*Y*] heteromeric models, this technique of model dimension reduction with a fast timescale is applied in an identical manner. In all cases, a new variable *X* is defined as the sum of every closed and open state, and then enforcing the fast equilibrium assumption results in an ODE of the form seen in Equation 4. The only differences between the QSS reductions for each of the different channels is the value of *N* and κ. We note here that this technique applies generally to models resembling any of these Markov schemes provided the assumptions about the kinetic rates (*a*_1_, *a*_2_, *b*_1_, *b*_2_ >> *a*_*I*_, *b*_*I*_) hold. Taking the QSS and full model versions of the *K*_*V*_1.1:*K*_*v*_1.4[*K*532*Y*] heteromeric and homomeric channels, we can compare the approximate and exact model solutions. This comparison is highlighted in Figure 6.

**Figure 7:**
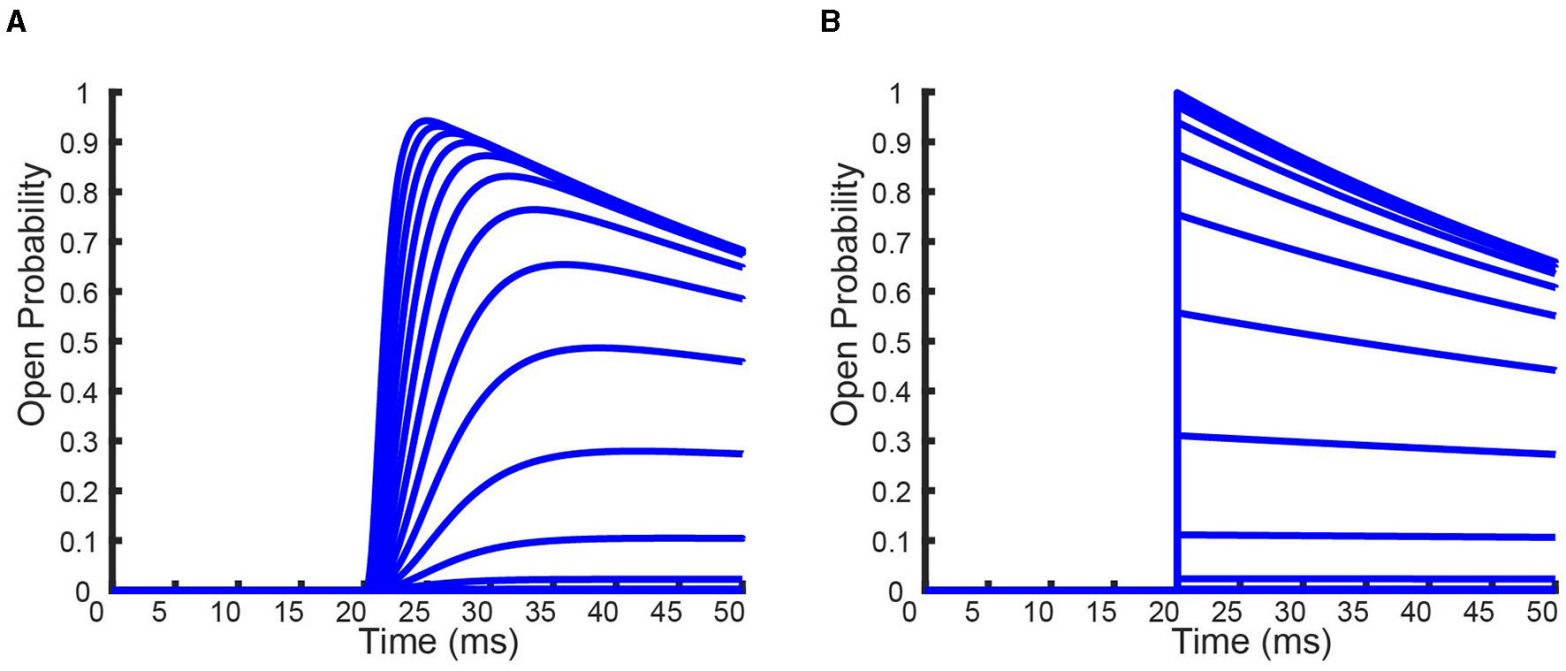
Full time simulations of the homomeric full **(A)** and QSS **(B)** *K*_*V*_1.4[*K*532*Y*] models in response to the activation protocol. Each curve denotes a different voltage that the channels has been held at.

In Figure 6 we see that for the inactivation and recovery protocols, the QSS model performs almost identically to the full model. The only noticeable difference between the models is in the response to the activation protocol. At voltage values greater than −30 *mV*, the QSS homomeric *K*_*V*_1.1:*K*_*v*_1.4[*K*532*Y*] models consistently predict a greater probability of opening than the full *K*_*V*_1.1:*K*_*v*_1.4[*K*532*Y*] models. The difference in models is best illustrated by looking at the full time simulated traces for the homomeric *K*_*v*_1.4[*K*532*Y*] channels in response to the activation protocol shown in Figure 7.

From Figure 7 it can be seen that the QSS model is immediately able to transition to its maximum probability of opening before inactivation takes place, which is forced by the QSS assumption. Whereas for the full model, the time it takes to reach its maximum probability of opening is long enough to allow for an increase in the probability of channel inactivation. The performance of this approximate solution on a more detailed voltage clamp protocol is provide in the Appendix. The detailed protocol is based on work by Fink and Noble (2009) and is also depicted in the Appendix.

### Homomeric C-type inactivating channels

The second form of inactivation to be modeled here, inherent to the α-protein subunits of *K*_*V*_ channels, is C-type inactivation. C-type inactivation, while less understood than N-type inactivation, is believed to result from conformational changes happening near the mouth of the pore or in the selectivity filter (Bett et al., 2011; Tan et al., 2022; Cuello et al., 2010). In the same 2011 study modeling N-type inactivation, Bett et al. (2011) also mutated *K*_1_.4 channels to delete the N-terminus ball (termed a Kv1.4ΔN channel) allowing them study and model C-type inactivation. This work led to a detailed Markov model that was able to replicate the responses of the Kv1.4ΔN channel to the protocols of Figure 1. Although this model was able to fit the experimental data and reflects the four transitions of each α-protein subunit opening, the biophysical mechanism of C-type channel inactivation is unclear based on the model structure.

**Figure 8:**
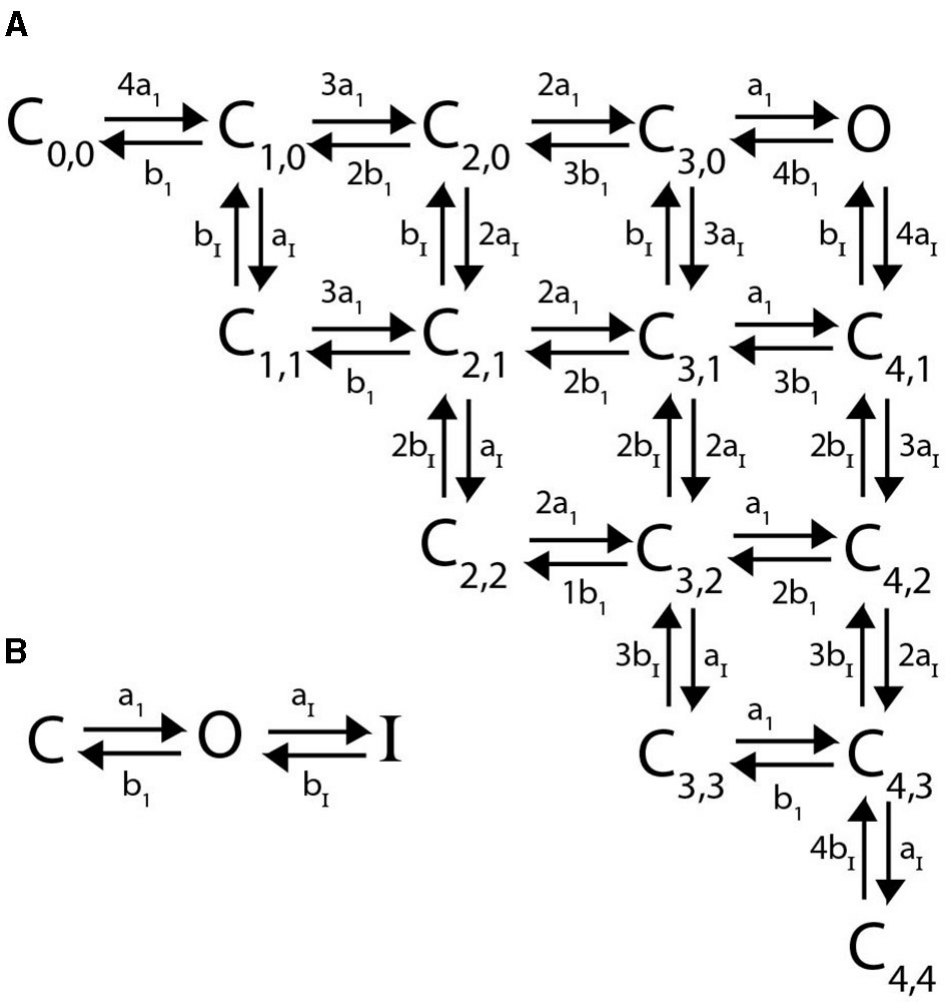
**(A)** A Markov model diagram for a homomeric channel with 4 C-type inactivating subunits. The state *C*_*i*_, *j* is the probability of having *i* subunits having reached the open state and *j* of these subunits being inactive. The state *O* is the conducting state where all 4 subunits are open and none are inactive. Rates *a*_1_, *b*_1_, *a*_*I*_, *b*_*I*_ are the corresponding forward and backward rates of a single C-type inactivating subunit transitioning between closed, open and inactive as is depicted in **(B)**.

We propose a new model structure where C-type inactivation is directly modeled as a conformational change of any of the four subunits from their open state. In particular, each of the four subunits can transition from a closed state *C* to an open state *O*, and only once it has reached an open state can it transition to an inactive state *I* (Figure 8B). Combining 4 identical, independent subunits that adhere to these kinetic transitions gives the full channel Markov model seen in Figure 8A, where the indices denote the number of subunits in the open and inactive states respectively.

### C-type inactivating model fitting

To justify working with this new model scheme, we must show that it is comparable in its ability to fit data to the (Bett et al., 2011) Kv1.4ΔN model. To do this, we first generated simulated data using the published Bett model and parameters in response to the three experimental protocols. Then for the scheme presented in Figure 8A, we create a system of ODEs according to the master equation given in Equation 5.


(5) $$\begin{array}{l} \begin{array}{c} \frac{dC_{i,j}}{dt}=-\left(4-i\right)a_{1}C_{i,j}-\left(i-j\right)b_{1}C_{i,j}+\left(4-i+1\right)a_{1}C_{i-1,j}\\ +\left(i+1\right)b_{1}C_{i+1,j}-\left(i-j\right)a_{I}C_{i,j}-jb_{i}C_{i,j}+\left(i-j+1\right)a_{I}C_{i,j-1}\\ +\left(j+1\right)b_{I}C_{i,j+1} \end{array} \end{array}$$

**Figure 9:**
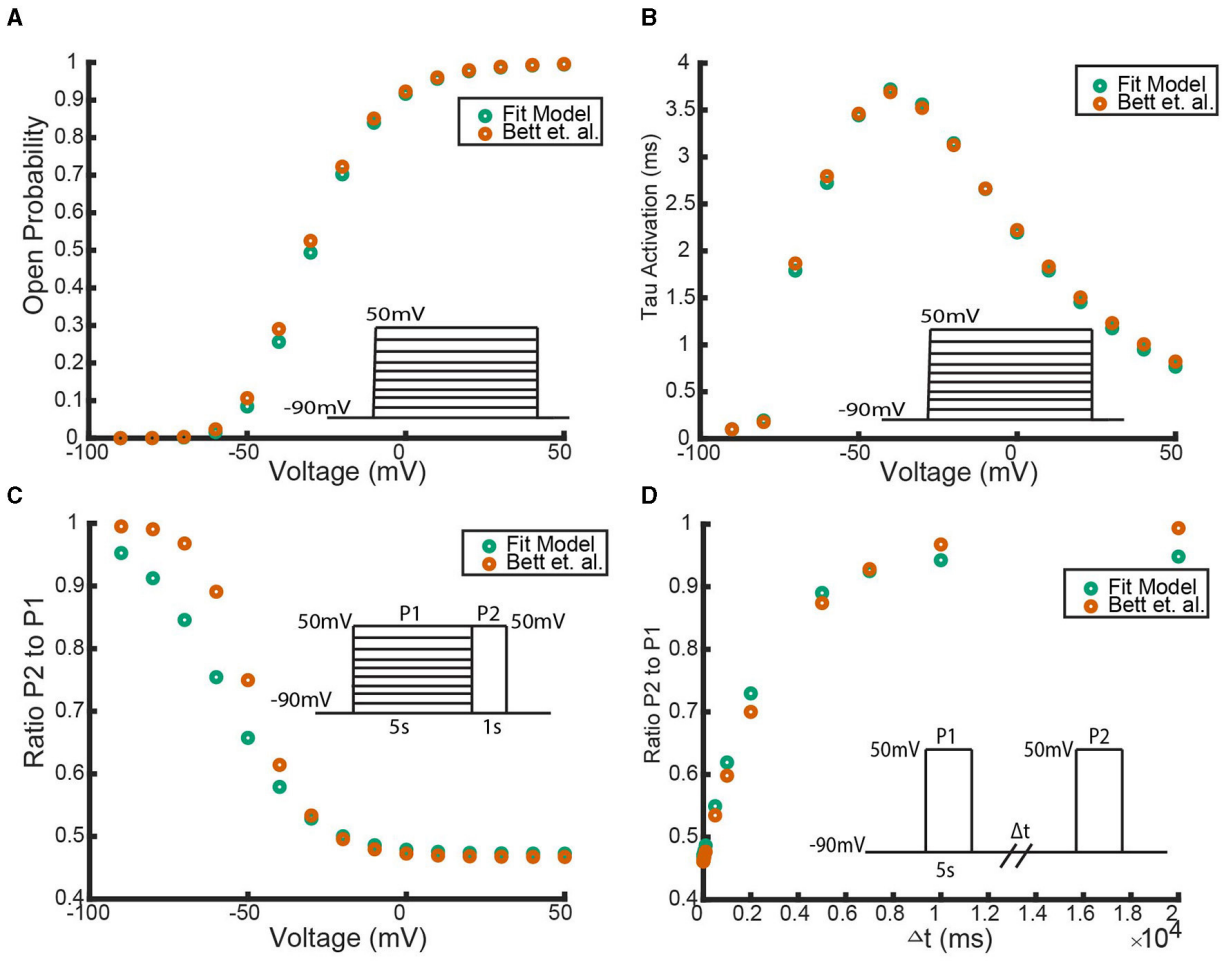
A comparison between the (Bett et al., 2011) simulated Kv1.4ΔN homomeric model and our hypothesized Kv1.4ΔN homomeric model. Four unique pieces of information were compared: **(A)** the open probability curves in response to the activation protocol, **(B)** the value of the activation time constant τ, **(C)** the response to the inactivation protocol, and **(D)** the response to the recovery protocol. The green data points are our model with the best fitting parameter set, and the orange data points are the (Bett et al., 2011) model. The inset diagrams depict the performed voltage clamp protocol.

As C-type inactivation is relatively voltage insensitive (Bett et al., 2011), the inactivation rates *a*_*I*_, *b*_*I*_ were set to be constant parameters, while *a*_1_, *b*_1_ were given the forms below in Equation 6


(6) $$\begin{array}{l} \begin{array}{c} a_{1}=m_{1}e^{n_{1}V}\\ b_{1}=m_{2}e^{n_{2}V} \end{array} \end{array}$$

for parameters *m*_1_, *n*_1_, *m*_2_, *n*_2_. To fit this model to the simulated (Bett et al., 2011) model data, we employed a bounded global parameter search on the simulated data. By minimizing the sum of squared errors between our model output and the Bett model output, we arrived at a parameter set that generated the model fit seen in Figure 9. For interested readers, more explicit parameter fitting details regarding the optimisation method, error function construction and parameter bounds are provided in the Appendix.

Figure 9 shows that with this parameter set our model can replicate the (Bett et al., 2011) model outputs. As there were a number of parameter sets found during the global parameter search that had a similar sum of squared errors, it is reasonable to hypothesize that a different parameter set for our model could fit the full experimental data as well.

### Heteromeric C-type inactivating channels

**Figure 10:**
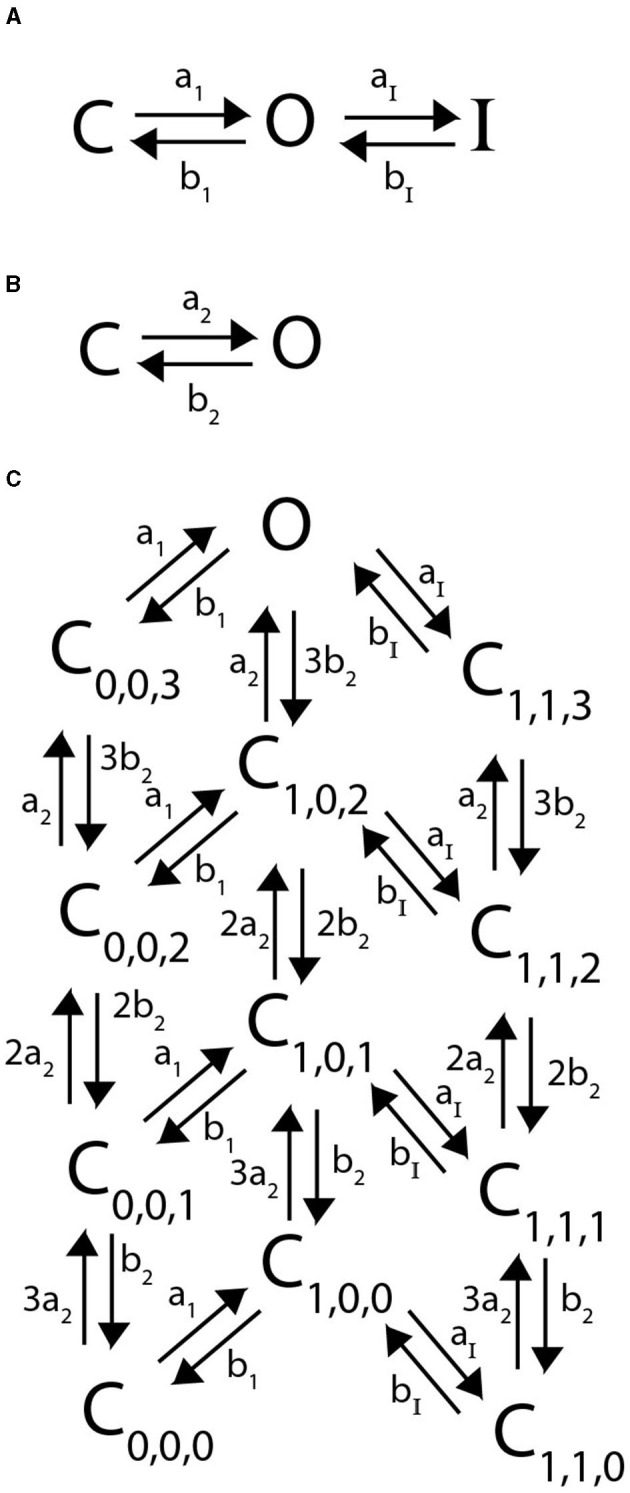
Markov model diagrams used to describe heteromeric channels with 1 C-type inactivating subunit and 3 non-inactivating subunits. Rates *a*_1_, *b*_1_, *a*_*I*_, *b*_*I*_ are the corresponding forward and backward rates of a single C-type inactivating subunit transitioning between closed, open and inactive as is depicted in **(A)**. Rates *a*_2_ and *b*_2_ are the corresponding forward and backward rates of a single non-inactivating subunit transiting between closed and open as is depicted in **(B)**. A Markov model diagram for a heteromeric channel with 1 C-type inactivating subunit and 3 non-inactivating subunits **(C)**. The state *C*_*i*_, *j, k* is the probability of having *i* C-type subunits having reached the open state, *j* C type subunits in the inactive state, and *k* non-inactivating subunits in the open state. The state *O* is the conducting state where all 4 subunits are in the openconformation and none are inactive.

Assuming that C-type inactivating subunits are modeled with the scheme presented in Figure 10A and non-inactivating subunits with the scheme shown in Figure 10B, we can construct any heteromeric combination of subunits. As with heteromeric non-inactivating channels and N-type inactivating channels; subunits of a specific type are assumed to be identical, all of a channel's subunits act independently, and all subunit kinetics are fit to the homomeric channels' voltage clamp experimental data. Again, provided these model assumptions hold, the framework is broadly applicable to other C-type inactivating subunits that are characterized mathematically in this manner. Presented in Figure 10C is the sample Markov diagram for a 3:1 heteromeric combination of 3 non-inactivating subunits and 1 C-type inactivating subunit. The Markov diagrams for the 2:2 and 1:3 heteromeric channels are provided in the Appendix in Figures 13, 14 for interested readers.

The corresponding master equations for any heteromeric combination of N non-inactivating subunits and M C-type inactivating subunits is given in Equation 7


(7) $$\begin{array}{l} \begin{array}{c} \frac{dC_{i,j,k}}{dt}=-\left(M-i\right)a_{1}C_{i,j,k}-\left(i-j\right)b_{1}C_{i,j,k}\\ +\left(M-i+1\right)a_{1}C_{i-1,j,k}+\left(i+1\right)b_{1}C_{i+1,j,k}\\ -\left(i-j\right)a_{I}C_{i,j,k}-jb_{i}C_{i,j,k}+\left(i-j+1\right)a_{I}C_{i,j-1,k}\\ +\left(j+1\right)b_{I}C_{i,j+1,k}+\left(N-k+1\right)a_{2}C_{i,j,k-1}+\left(k+1\right)b_{2}C_{i,j,k+1}\\ -\left(N-k\right)a_{2}C_{i,j,k}-kb_{2}C_{i,j,k}. \end{array} \end{array}$$

**Figure 11:**
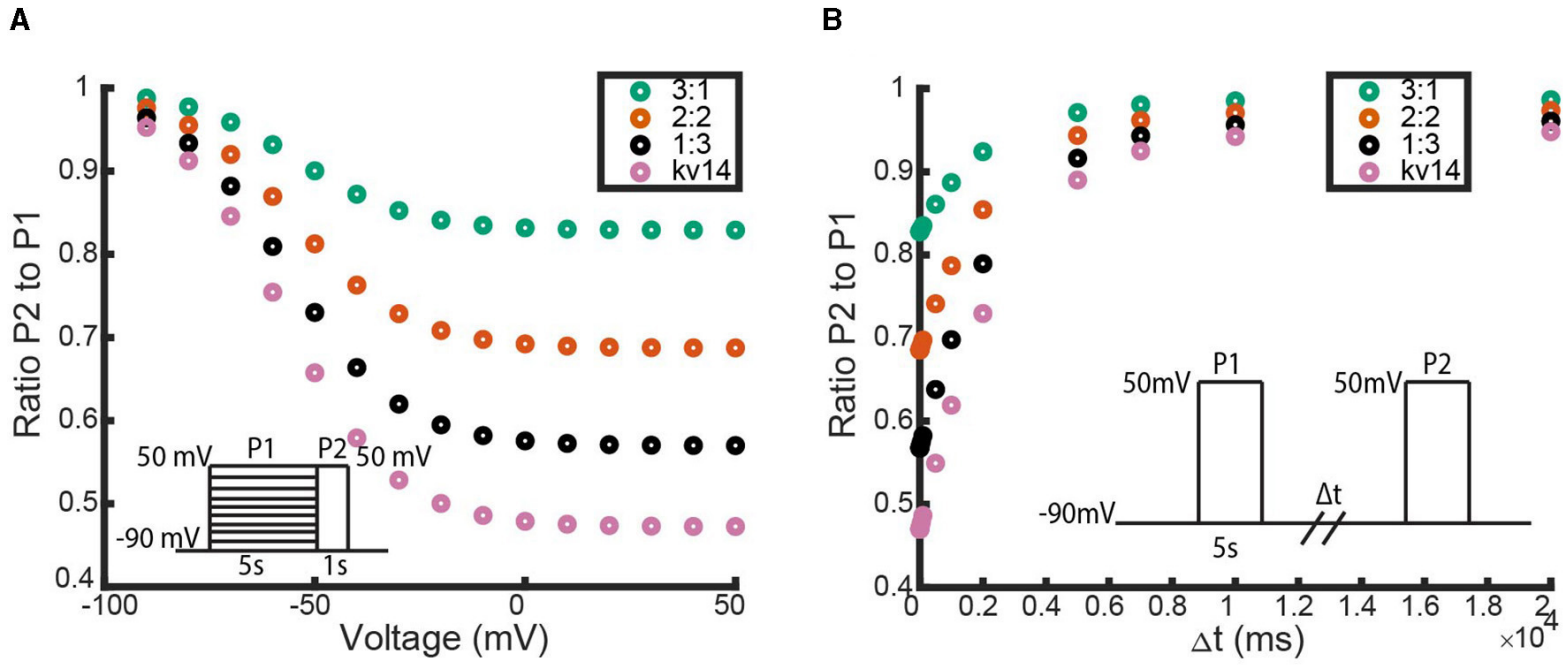
Responses of each heteromic and homomeric channel with *K*_*V*_1.4ΔN subunits and *K*_*V*_1.1 subunits to the inactivation and recovery voltage clamp protocols (Figure 1). **(A)** Identical plot meaning and data point coloring to that of Figure 4A, but with our *K*_*V*_1.1:*K*_*V*_1.4ΔN models. **(B)** Identical plot meaning and data point coloring to that of Figure 4B, but with our *K*_*V*_1.1:*K*_*V*_1.4ΔN models.

As with the N-type models, we explore the impact of adding in additional inactivating subunits by simulating each heteromeric channel model through the three voltage clamp protocols depicted in Figure 1. For the homomeric non-inactivating kinetics we again work with a *K*_*V*_1.1 channel parameterized with transition rates from Masoli et al. (2015). For C-type inactivation, we use the parameter set generated by the fitting methodology outlined in “Section: C-Type Inactivating Model Fitting” for the *K*_*V*_1.4ΔN channels. The simulation results for the inactivation and recovery voltage clamp protocols across all heteromeric *K*_*V*_1.1:*K*_*V*_1.4ΔN channels are summarized in Figure 11.

In Figure 11, there are two immediate observations to make. The first observation is that like the non-inactivating:N-type heteromers, a single *K*_*V*_1.4ΔN subunit is sufficient to supply some, albeit small, amount of channel inactivation (Figures 11B, C). The second noticeable feature is that as the number of *K*_*V*_1.4ΔN subunits is increased, the level of inactivation is increased and the rate of recovery is decreased. However, unlike with the non-inactivating:N-type heteromeric channels, this relationship between *K*_*V*_1.4ΔN subunits and inactivation appears to be almost linear. Each additional *K*_*V*_1.4ΔN subunit roughly confers an equal amount of inactivation and has a similar impact on the time to recovery.

### C-type inactivating model: invariant manifold

As was noted in the previous section, the dimension of the non-inactivating:C-type heteromeric channel models becomes quite large (smallest model is 12 ODEs). For heteromeric channel models described by the master equation seen in Equation 7, we show there is a 3 dimensional invariant manifold which stems from the assumption of identical and independent subunits. Using some results from Keener (2009, 2010) we detail the proof for these invariant manifold reductions using the 2:2 heteromer as an example.

Based on Keener (2009), the binomial distribution gives a vector *P*_*k*_ describing the probability of having *k* open, non-inactivating subunits. This vector


(8) Pk=(2k)qk(1-q)k

is an invariant manifold for the set of equations which describe the interactions between 2 non-inactivating subunits. This holds provided the parameter *q*, the probability of a single subunit being in the open state, satisfies the ODE:


(9) $$\begin{array}{l} \frac{dq}{dt}=a_{2}\left(1-q\right)-b_{2}q. \end{array}$$

We can find an invariant manifold *P*_*i,j*_, for 2 interacting C-type subunits, which describes the probability of having *i* C-type subunits having reached the open state and *j* C-type subunits in the inactive state. This invariant manifold is given by the multinomial distribution:


(10) $$\begin{array}{l} P_{i,j}=\frac{2!}{\left(2-\left(i-j\right)\right)!\left(i-j\right)!j!}{\left(1-n-h\right)}^{2-\left(i-j\right)}n^{i-j}h^{j}. \end{array}$$

so long as the parameters *n* (probability of a single subunit being open), and *h* (probability of an open subunit being inactive) satisfy


(11) $$\begin{array}{l} \begin{array}{c} \frac{dn}{dt}=-\left(b_{1}+a_{I}\right)n+a_{1}\left(1-n-h\right)+b_{I}h\\ \frac{dh}{dt}=a_{I}n-b_{I}h. \end{array} \end{array}$$

Note that for either type of subunit, the invariant manifold dimension is 1 less than the number of subunit states as a result of working with probabilities. For example, with the non-inactivating subunits, if *q* describes the probability of a single subunit being open, then (1 − *q*) gives the probability of the subunit being closed. Similarly, for C-type inactivating subunits, if we track the probability of a single subunit being open (*n*) or inactive (*h*), then the probability that subunit is closed is (1 − *n* − *h*).

If Equations 8, 10 give invariant manifolds for 2 non-inactivating subunits and 2 C-type inactivating subunits respectively, we should have that $P_{i,j,k}=P_{k}^{T}P_{i,j}$ is an invariant manifold for Equation 7 with *N* = *M* = 2. To prove this, we begin by rewriting Equation 7 as


(12) $$\begin{array}{l} \frac{dP}{dt}=RP \end{array}$$

where P is an 18 dimensional state vector, and *R* is the corresponding 18 × 18 rate transition matrix. We show that *P*_*i,j,k*_, with the parameter dynamics of Equations 9, 11, is an invariant manifold for Equation 7 by demonstrating that


(13) $$\begin{array}{l} RP_{i,j,k}=\frac{\partial P_{i,j,k}}{\partial q}\frac{dq}{dt}+\frac{\partial P_{i,j,k}}{\partial n}\frac{dn}{dt}+\frac{\partial P_{i,j,k}}{\partial h}\frac{dh}{dt} \end{array}$$

The left hand side of this equality should be thought of as trajectories in the entire space being evaluated on the manifold, while the right hand side gives trajectories of the reduced space. Showing equality implies that once the full 18 dimensional system reaches *P*_*i,j,k*_, it is restricted to movement along *P*_*i,j,k*_, giving us an invariant manifold. Proof of this equality is a matter of matrix multiplication which is left to the reader. However as a guide, here we show a single row's worth of calculations using the 18^*th*^ row of *R*.


(14) $$\begin{array}{l} \begin{array}{c} R\left(18\right)P_{i,j,k}\\ =-\left(2b_{I}+2b_{2}\right)q^{2}h^{2}+a_{I}\left(2q^{2}nh\right)+a_{2}\left(2q\left(1-q\right)h^{2}\right)\\ =2qh^{2}\left(a_{2}\left(1-q\right)-b_{2}q\right)+2q^{2}h\left(a_{I}n-b_{I}h\right)\\ =\frac{\delta P_{i,j,k}}{\delta q}\frac{dq}{dt}+\frac{\delta P_{i,j,k}}{\delta h}\frac{dh}{dt}. \end{array} \end{array}$$

Explicit expressions for *P*_*i,j,k*_ and the rate transition matrix *R* are provided in the Appendix.

It is reemphasized here that reducing the full Markov scheme in this manner provides an exact, and not approximate, simplified model. For this reason, no comparison simulations between the full and reduced models, as was done in Figure 6 for the N-type QSS model, are required. All future simulations and work can be conducted with the new reduced model and will provide identical results in tracking the open probability. However, the full Markov scheme still provides useful understanding by framing the model in terms of its physiological context as it relates to the opening, closing and inactivating of the α-subunits.

## Discussion

In our previous work (McGahan and Keener, 2022), we showed how to translate from the standard Markov model for a homomeric channel with 4 identical, non-inactivating subunits to a heteromeric channel with 2 different types of non-inactivating subunits. Here we built off of this work by first modeling channel inactivation in a homomeric channel, and then by showing how subunits from an inactivating channel could be incorporated into a heteromeric channel model. In this study we constructed homomeric and heteromeric models for channels possessing either of the two primary inactivation mechanisms: N-type and C-type inactivation. In both cases, the homomeric models were based on experimental data, and the heteromeric models were then a result of the model assumptions with no further fitting. In spite of the limited heteromeric experimental work within the literature to date, we show (and discuss) which known properties our models reproduce and then highlight each of the testable predictions we were able to produce from our models.

### N-Type inactivation

The model we used for a homomeric N-type inactivating channel, shown in Figure 2, was identical to the model presented by Bett et al. (2011). This model structure nicely reflects the known experimental observations that there are 4 identical subunits which must change to the open conformation to enter the conducting state, that only a single N-terminal “ball” can bind to the open channel state, and that this binding is a voltage insensitive transition.

Working with this homomeric N-type inactivating channel model, we described the manner by which subunits of this type would form heteromeric complexes with subunits of a non-inactivating channel. The heteromeric channels were similarly required to have 4 activation steps, with number and type identical to subunit number and type, and with a number of N-balls equal to the number of N-type subunits (Figure 3). To examine the predictions made by our proposed heteromeric model structure, we found fully parameterized homomeric models, for both N-type inactivating and non-inactivating channels that are known to form heteromers (Harding et al., 2022; Masoli et al., 2015; Bett et al., 2011). By simulating each homomeric and heteromeric channel through the three different voltage clamp protocols shown in Figure 1, we observed two key properties of this model.

The first result of this model, which is in agreement with known experimental evidence, is that a single N-type subunit is sufficient to confer some channel inactivation (Figures 4B, C) (Coetzee et al., 1999; MacKinnon et al., 1993). The second predictive result of this model, is that while increasing the number of N-type subunits does increase the level of inactivation, each additional N-type subunit has a diminished impact (Figure 4). This non-linear effect is similar to what has been observed and modeled with the changes in open probability curves of non-inactivating *K*_*V*_1.1:*K*_*V*_1.2 heteromers in response to increasing the number of *K*_*V*_1.2 subunits (McGahan and Keener, 2022; Al-Sabi et al., 2013). In the case of the non-inactivating *K*_*V*_1.1:*K*_*V*_1.2 channels, the non-linearity is a result of needing all four subunits to be in the open state. This means the channel opening is rate limited by the last subunit, in this case *K*_*V*_1.2, to open. With the N-type:non-inactivating heteromers, the non-linearity observed in the inactivation and recovery protocols can also be explained as a rate-limited effect. With this model, as the number of N-type inactivating subunits is increased, so is the number of N-balls. However, given that only one ball can bind to the pore opening at a given time, there is a competitive binding site saturation for the N-type balls, where adding additional N-balls eventually stops increasing the overall rate at which the first one of them can bind to the pore.

Despite the current experimental complications with studying inactivating heteromeric channels, and thus limited literature on the topic, there is one particularly relevant study to our N-type model done by Hashimoto et al. (2000) looking at modified *K*_*V*_1.4 channels. In their work they were able to construct versions of a *K*_*V*_1.4 channel possessing either 1, 2, or 4 tethered N-balls. The key result, as it relates to our model, was an identical appearance of a non-linear effect of increasing the numbers of N-balls. More precisely, they show that the inactivation rate of a channel with 2 balls is more than half that of a 4 balled channel, and the inactivation rate of a 1 balled channel is more than one fourth that of the full homomeric *K*_*V*_1.4 channel. Additionally, their experiments reveal that as the number of inactivation balls is decreased the level of channel inactivation became less complete. In tandem, these results create a nice qualitative match with our model output given in Figure 4. Finally, Hashimoto et al. (2000) also provides a biological mechanism behind the non-linear impact of each N-ball and the incomplete inactivation by hypothesizing that an electrostatic repulsive force felt between the balls is contributing to the decreased effectiveness of N-ball binding.

### C-Type inactivation

In their 2011 work, Bett et al. (2011) published a homomeric C-type inactivating model that was fit to experimental voltage clamp data. While their model was able to fit the data well, the chosen model structure does not help capture or explain the biophysical mechanisms of C-type inactivation that are at play. We proposed a new homomeric C-type model that encodes the C-type conformational change into the model structure, by assuming each of the 4 subunits has a closed, open, and inactive state (Figure 8). To justify using this new model, we first generated data by simulating the published (Bett et al., 2011) model for *K*_*V*_1.4Δ*N* channels through the three voltage clamp protocols from Figure 1. Then by performing a bounded, global parameter search we showed that there is at least one parameter set which qualitatively has the same properties as the (Bett et al., 2011) model (Figure 9). We note that while this may not be the optimal fitting parameter regime, with the limited available data we were still able to qualitatively match the channel behavior.

Utilizing our C-type inactivating homomeric model, we were able to construct heteromeric channels with C-type inactivating *K*_*V*_1.4Δ*N* channels and non-inactivating *K*_*V*_1.1 channels. The only assumption we needed to make was that each heteromeric channel has 4 total independent subunits, which individually are modeled with the ODEs corresponding to Figure 10A (C-type subunits), or Figure 9B (non-inactivating subunits). Taking each possible combination of these subunit types, we simulated the channels through the voltage clamp protocols and highlighted the response of these channels to the inactivation and recovery protocols (Figure 11). Like the N-type:non-inactivating model, these simulations revealed that a single *K*_*V*_1.4Δ*N* subunit was enough to make the heteromeric channel inactivate. Furthermore, as the number of C-type subunits is increased, the level of inactivation and time to recovery are increased.

However, in contrast to the N-type:non-inactivating model, this change in inactivation and rate of recovery scales linearly with the number of C-type inactivating subunits. Unlike the inactivation of heteromers with N-type inactivating subunits, there is no binding saturation issue since the inactivation is a conformational change inherent to the individual subunits, which are operating independently of one another. This independence of inactivation is what gives the linear relationship between number of C-type inactivating subunits and level of inactivation.

### Implications of inactivation differences

A unique capability of this modeling study is that it allows us to compare between heteromeric channels with N versus C type inactivating subunits. One way to illuminate the similarities and differences is by framing them in the context of ion channel viability. In particular, which combinations of subunits would one expect to see naturally expressed, and which of these combinations could actually be functional channels? One possible hypothesis we propose based on these results is that heteromeric channel viability could be a result of how distinct their kinetic properties, and thus influence on spike timing and dynamics, are from their homomeric counterparts.

The first point reemphasized here is that all heteromeric channels with an inactivating subunit will inactivate thereby distinguishing them from a non-inactivating channel. That being said, there is one glaring difference between subunit types. Due to the linear effects of adding additional C-type inactivating subunits discussed in the previous section, there would be a wider degree of variability among the heteromeric properties than for N-type inactivating heteromers. If all heteromers with inactivating subunits are viable, then those with C-type subunits would cover a wider kinetic range thus markedly changing the window of cellular excitability from a cell containing only homomeric channels. Meanwhile, the N-type inactivating heteromers look remarkably similar to each other in response to the voltage clamp protocols regardless of the number of N-type subunits. Therefore, assuming the N-type inactivating homomer is functional, one might expect these heteromeric channels to be equally viable since interchanging N-type subunits with non-inactivating subunits likely produces indistinguishable neuronal dynamics. Future concatameric and co-expression experiments like the studies by Al-Sabi et al. (2011, 2013) and Cordeiro et al. (2019) will be critical for answering these questions surrounding the functionality of heteromeric channels of different subunit ratios.

### Model dimension reduction

The final outcome of this work stemmed from examining the model dimension of our various heteromeric channels. Although the smallest model, the N-type homomeric channel, consists of only 6 ordinary differential equations, the largest C-type:non-inactivating heteromeric channel model has 20 differential equations. Individually, any one of these channel models is small enough to include in a full neuron model for simulation. That being said, a common experimental procedure where mathematical modeling has been found useful, is one where two different subunit DNAs are injected into a cell and the resulting distribution of the heteromeric and homomeric channels that form is unknown (McGahan and Keener, 2022; D'Adamo et al., 1999; Miceli et al., 2013, 2015; Hasan et al., 2017). To compare to experiments of this type, and gain an understanding of the types of heteromeric channels that are forming as a result of this procedure, requires modeling every possible subunit combination. Doing so would necessitate having 39 ODEs for every N-type:non-inactivating channel and 70 ODEs to include every C-type:non-inactivating channel.

Recall that the form and size of these models is enforced by the physiologically grounded model assumptions and not due to phenomenological construction. Therefore, our best option for making these models more computationally tractable, while continuing to adhere to the biological assumptions that require these model structures, is to consider possible model dimension reduction techniques.

For the N-type:non-inactivating heteromeric channels, since the presence of the single inactive state eliminates the possibility of finding a nice globally stable invariant manifold, we found Quasi-Steady State approximations of the full models. By assuming the transitions between closed states are faster than the transition between the open and inactive state, which we validated by looking at experimentally fit rate constants (Figure 5), we showed that each heteromeric N-type:non-inactivating model could be reduced to 2 ODEs (Equation 4). Comparing the results of the full and reduced models showed that the Quasi-Steady State approximation almost exactly replicates the full models performance for the inactivation and recovery protocols (Figure 6). The only noticeable distinction between our full and reduced models is their predictions of open probability curves in response to the activation protocol. The QSS model consistently overestimated the probability of being open in comparison to the full model (Figure 7). This is a consequence of assuming the activation is happening on a fast enough timescale that channels are fully able to open before any inactivation takes place.

For the C-type:non-inactivating heteromeric channels we showed that each model has a globally stable invariant manifold of reduced dimension. Based on a result of Keener (2009, 2010), the homomeric C-type channel model (Equation 5) has a two dimensional invariant manifold given by a multinomial distribution. Additionally, work by Keener (2009, 2010), has shown that the non-inactivating channel (Equation 1) has an invariant manifold given by a binomial distribution, where the open probability is exactly described by the equations of the Hodgkin-Huxley potassium channel (Hodgkin and Huxley, 1952). By using the multiplicative structure inherent to invariant manifolds, we demonstrate that each heteromeric C-type:non-inactivating channel has a 3 dimensional invariant manifold which is the product of the corresponding multinomial and a binomial distributions.

### Future model uses

While one limitation of this work was a lack of data and existing experimental literature to compare with, this limitation also highlights our model's strengths and at possible future applications. Our model provides a biologically relevant story behind the entire class of less characterized, but highly prevalent, heteromeric ion channels. As lab techniques for future study of heteromeric channels become available; this study can be used to guide predictions, probe experimental outputs, and frame these outputs in terms of the intuition gained from our biophysical model. One immediate application this model is suited for is the examination of coexpression experiments (D'Adamo et al., 1999; Miceli et al., 2013, 2015; Hasan et al., 2017) to see if there are preferred functional stoichiometries of inactivating and non-inactivating subunits. As detailed in McGahan and Keener (2022), this model formulation, especially the reduced versions, provides an easy pathway to examine the distribution of subunits in inactivating *K*_*V*_ channels to see if there is either heteromeric or homomeric preference, or a tendency toward assembly according to a binomial distribution.

A second direction for future exploration and modeling of heteromeric channels involves the existence of heteromeric channels formed with auxiliary beta subunits and pore-forming alpha subunits. While we emphasize here that it is crucial to understand the types of heteromers formed from only alpha subunits, it is also well known that in some *K*_*V*_ families there are auxiliary beta subunits that can alter channel properties. The strength of these alterations are dependent on the number of beta subunits, and can take the form of introducing inactivation to an otherwise non-inactivating channel or shifting the activation curves of the pore-forming subunits (England et al., 1995; Pongs and Schwarz, 2010; Heinemann et al., 1996; Xu et al., 1998). In the case of introducing an auxiliary subunit which confers inactivation upon the pore-forming subunits (Xu et al., 1998), there is a direct parallel to our Heteromeric N-type inactivating model. For example, a Markov model with 4 non-inactivating alpha subunits and 4 inactivating beta subunits would mimic (Figure 2) where the transition to the I state would now represent the auxiliary beta subunits instead of the N-balls. On the other hand, auxiliary subunits known for shifting activation curves of the pore-forming channel are likely a more complex phenomenon to model as this type of interaction necessarily implies some form of dependence between subunit state transitions (Pongs and Schwarz, 2010; England et al., 1995). Once the assumption of independence is violated, these Markov style models would no longer apply, and would therefore require a new model framework.

## Conclusion

Here we have proposed a new model for heteromeric channels which are constructed from C/N-type inactivating subunits and non-inactivating subunits. This hypothesized model structure allowed us to make novel testable predictions about the relationship between the number of subunits that confer inactivating kinetics and the level of channel inactivation. These models demonstrated a noticeable difference between the types of inactivation (N versus C type) and the strength of heteromeric channel inactivation. Moving forward, these models can help frame the approach future researchers take to investigate heteromeric channel kinetics, particularly as it relates to channel inactivation.

## Data Availability

Publicly available datasets were analyzed in this study. This data can be found here: https://github.com/keesmcgahan/Heteromeric-Inactivating-KChannels.
