# Supplementary material for: Mathematical models of C-type and N-type inactivating heteromeric voltage gated potassium channels
Source: Front Cell Neurosci. 2024 Oct 8;18:1418125. doi: 10.3389/fncel.2024.1418125 (PMC11493646; doi:10.3389/fncel.2024.1418125)
Supplement: Supplementary file 1 [file Data_Sheet_1.pdf]

APPENDIX

QSS to Full Comparison

Here in Supplemental Figure 1 we show the performance of our approximate QSS solution on a more detailed voltage clamp protocol. The protocol is a condensed version of a protocol presented in work by (Fink and Noble, 2009). The protocol is show in Supplemental Figure 1 A. while the response of the QSS and full N-type homomeric models are shown in Supplemental Figure 1 B. The goal of this protocol is to expose the channel models to voltages where they would experience activation, inactivation, deactivation and reactivation Fink and Noble (2009).

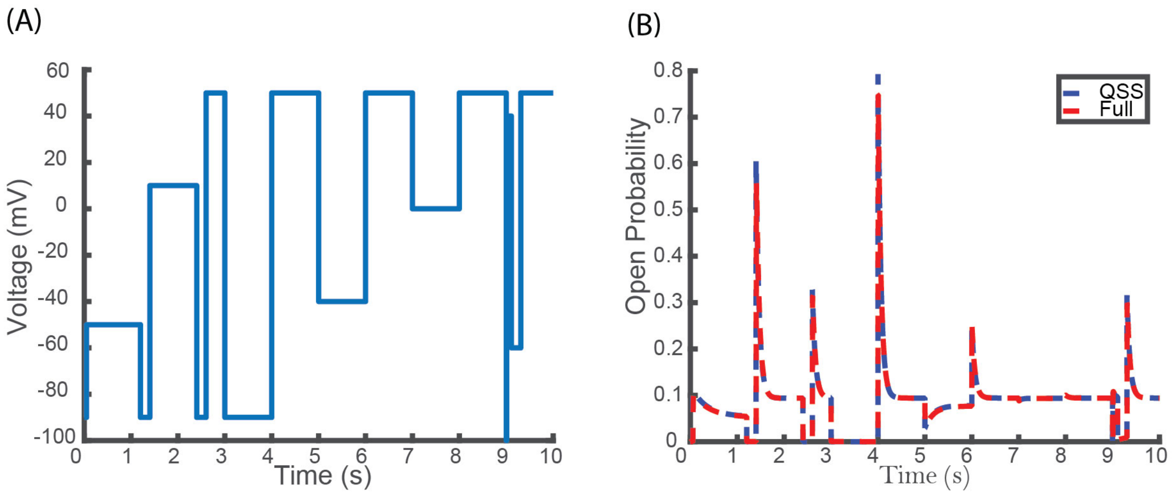

Supplemental Figure 1. Time simulations **(B)** of the full (red) and QSS (blue) approximate homomeric $K_V 1.4[K532Y]$ models in response to a detailed voltage clamp protocol **(A)**. The protocol is a slightly altered version of a setup detailed in (Fink and Noble, 2009).

Parameter Fitting

The original C-type Bett model (Bett et al., 2011) was based on data from (Comer et al., 1994). Using the best fitting C-type Bett model we generated simulated data vectors for each of the three experimental protocols seen in Figure 1 in the manner detailed in the Methods section. We then perform a bounded global parameter optimization search on the homomeric model scheme shown in Figure 8.

The first thing specified to complete this search is the parameter bounds. In this case, the rate equations in Eq. 6 are defined such that all parameters have non-negative values. Upper bounds were given limits only because the overall rates a_1 and b_1 could reach exponential blowup at the extreme voltage values otherwise. Parameters, and their upper bounds, were then normalized for algorithm efficiency and to provide identical weighting to each parameter during error function calculation. Following this, we employ Matlab's (MATLAB, 2018) genetic algorithm (GA) package to search the parameter space for possible parameter sets. The GA is a global search method (Lambora et al., 2019) whose simplified details are listed as follows:

1. It selects multiple initial parameter sets randomly.
2. It performs gradient descent using these initial parameter sets where the gradient is defined via some objective function.

3. It eliminates badly performing parameter sets and replaces them with parameter sets that have randomly altered and combined with parameter values from previous iterations.

The objective function used here to perform gradient descent consisted of four equally weighted terms. Each term is a sum of squared errors between the Bett model and our Homomeric C-type model when simulated through the voltage clamp protocols (Figure 1). The data generated from these simulations correspond to: activation open probability, activation time constant τ, level of inactivation, and recovery from inactivation. Details for the specific calculation of each of these data types is made clear in the methods section in the primary document in tandem with Figure 1.

C-type Heteromer Details

The other two C-type inactivating heteromeric channel markov models are provided here for reader clarity. Supplemental Figure 2 depicts the heteromeric channel with 2 C-type inactivating subunits and 2 non-inactivating subunits. In Supplemental Figure 3 the proposed markov model for a heteromeric channel with 3 C-type inactivating subunits and 1 non-inactivating subunits is shown.

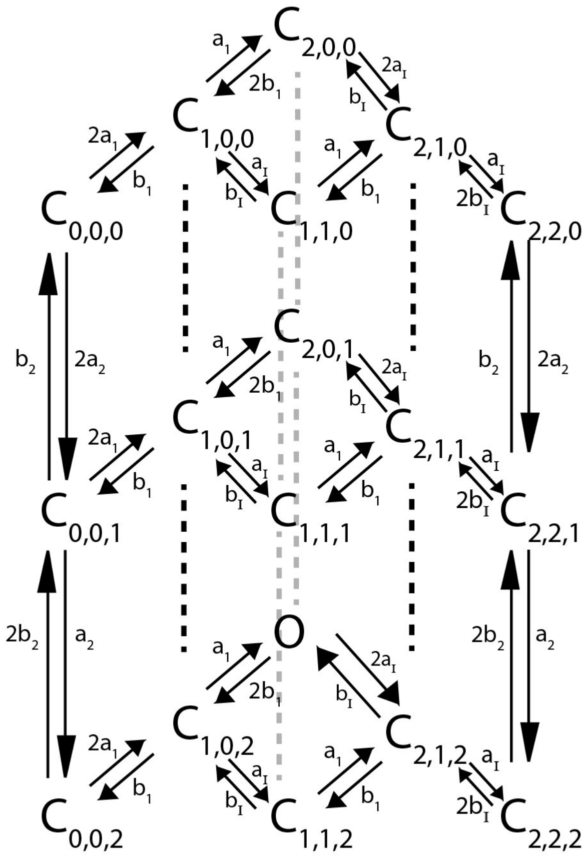

Supplemental Figure 2. Markov model diagram used to describe heteromeric channels with 2 C-type inactivating subunits and 2 non-inactivating subunits. The state C_i, j, k is the probability of having i C-type subunits having reached the open state, j C type subunits in the inactive state, and k non-inactivating subunits in the open state. The state O is the conducting state where all 4 subunits are in the open conformation and none are inactive. Rates a_1, b_1, a_I, b_I are the corresponding forward and backward rates of a single C-type inactivating subunit transitioning between closed, open and inactive as is depicted in Figure 10A. Rates a_2 and b_2 are the corresponding forward and backward rates of a single non-inactivating subunit transiting between closed and open as is depicted in Figure 10B

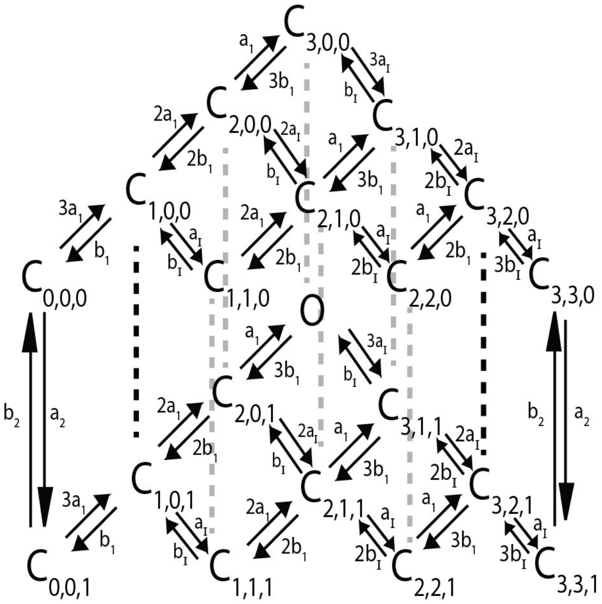

Supplemental Figure 3. Markov model diagram used to describe heteromeric channels with 3 C-type inactivating subunits and 1 non-inactivating subunits. The state C_i, j, k is the probability of having i C-type subunits having reached the open state, j C type subunits in the inactive state, and k non-inactivating subunits in the open state. The state O is the conducting state where all 4 subunits are in the open conformation and none are inactive. Rates a_1, b_1, a_I, b_I are the corresponding forward and backward rates of a single C-type inactivating subunit transitioning between closed, open and inactive as is depicted in Figure 10A. Rates a_2 and b_2 are the corresponding forward and backward rates of a single non-inactivating subunit transiting between closed and open as is depicted in Figure 10B

C-type Invariant Manifold details

The matrices for the C-type Inactivating Model invariant manifold derivation are given below:

$$Q = \begin{pmatrix} -2a_1 & b_1 & 0 & 0 & 0 & 0 \\ 2a_1 & -b_1 - a_1 - a_I & 2b_1 & b_I & 0 & 0 \\ 0 & a_1 & -2b_1 - 2a_I & 0 & b_I & 0 \\ 0 & a_I & 0 & -b_I - a_1 & +b_1 & 0 \\ 0 & 0 & 2a_I & a_1 & -b_I - b_1 - a_I & 2b_I \\ 0 & 0 & 0 & 0 & a_I & -2b_I \end{pmatrix}$$

$$B = \begin{pmatrix} b_2 & 0 & 0 & 0 & 0 & 0 \\ 0 & b_2 & 0 & 0 & 0 & 0 \\ 0 & 0 & b_2 & 0 & 0 & 0 \\ 0 & 0 & 0 & b_2 & 0 & 0 \\ 0 & 0 & 0 & 0 & b_2 & 0 \\ 0 & 0 & 0 & 0 & 0 & b_2 \end{pmatrix}$$

$$A = \begin{pmatrix} a_2 & 0 & 0 & 0 & 0 & 0 \\ 0 & a_2 & 0 & 0 & 0 & 0 \\ 0 & 0 & a_2 & 0 & 0 & 0 \\ 0 & 0 & 0 & a_2 & 0 & 0 \\ 0 & 0 & 0 & 0 & a_2 & 0 \\ 0 & 0 & 0 & 0 & 0 & a_2 \end{pmatrix}$$

$$R = \begin{bmatrix} Q-2A & B & 0 \\ 2A & Q-A-B & 2B \\ 0 & A & Q-2B \end{bmatrix}$$

$$P_{i,j,k} = \begin{pmatrix} (1-q)^2(1-n-h)^2 \\ 2(1-q)^2(1-n-h)n \\ (1-q)^2n^2 \\ 2(1-q)^2h(1-n-h) \\ 2(1-q)^2nh \\ (1-q)^2h^2 \\ 2q(1-q)(1-n-h)^2 \\ 2q(1-q)(1-n-h)n \\ 2q(1-q)n^2 \\ 4q(1-q)h(1-n-h) \\ 2q(1-q)nh \\ 2q(1-q)h^2 \\ q^2(1-n-h)^2 \\ 2q^2(1-n-h)n \\ q^2n^2 \\ 2q^2h(1-n-h) \\ 2q^2nh \\ q^2h^2 \end{pmatrix}$$

REFERENCES

Bett, G. C., Dinga-Madou, I., Zhou, Q., Bondarenko, V. E., and Rasmusson, R. L. (2011). A model of the interaction between n-type and c-type inactivation in kv1. 4 channels. *Biophysical journal* 100, 11–21

Comer, M. B., Campbell, D. L., Rasmusson, R. L., Lamson, D. R., Morales, M. J., Zhang, Y., et al. (1994). Cloning and characterization of an ito-like potassium channel from ferret ventricle. *American Journal of Physiology-Heart and Circulatory Physiology* 267, H1383–H1395

Fink, M. and Noble, D. (2009). Markov models for ion channels: versatility versus identifiability and speed. *Philosophical Transactions of the Royal Society A: Mathematical, Physical and Engineering Sciences* 367, 2161–2179

Lambora, A., Gupta, K., and Chopra, K. (2019). Genetic algorithm-a literature review. In *2019 international conference on machine learning, big data, cloud and parallel computing (COMITCon)* (IEEE), 380–384

MATLAB (2018). *9.7.0.1190202 (R2019b)* (Natick, Massachusetts: The MathWorks Inc.)
